# Evidence-based medicine and values-based medicine: partners in clinical education as well as in clinical practice

**DOI:** 10.1186/1741-7015-11-40

**Published:** 2013-02-15

**Authors:** Ed Peile

**Affiliations:** 1 Warwick Medical School, The University of Warwick, Coventry, CV4 7AL, UK

**Keywords:** clinical decision making, complexity, conflict, evidence-based medicine, values-based medicine

## Abstract

The best clinical decisions are based on both evidence and values in what is known as the 'two-feet principle'. Anecdotally, educationalists find teaching clinicians to become more evidence based is relatively simple in comparison to encouraging them to become more values based. One reason is likely to be the importance of values awareness. As values-based practice is premised on a mutual respect for the diversity of values, clinicians need to develop the skills to ascertain patient values and to get in touch with their own beliefs and preferences in order to understand those at play in any consultation. Only then can shared decision-making processes take place within a shared framework of values. In a research article published in *BMC Medicine*, Altamirano-Bustamante and colleagues highlight difficulties that clinicians face in getting in touch with their own values. Despite finding that healthcare personnel's core values were honesty and respect, autonomy was initially low ranked by participants. One significant aspect of this work is that this group has demonstrated that the extent to which clinicians value 'autonomy' and 'openness to change' can both be positively influenced by well designed education.

## Introduction

Evidence-based medicine (EBM) and values-based medicine (VBM) are complementary partner components of clinical decision making. This has been referred to as the 'two-feet principle' [1]. Both at the level of diagnosis and for management of a clinical problem it is important that clinicians can work comfortably and effectively with scientific evidence relevant to patient's problem, and with the values at play which comprise not only the patient's values but also the clinician's values and often those of others such as carers or the healthcare organization [2]. Values can be complex and conflicting [3], and it is here that the skills of values-based practice are needed if shared decision making is to happen within a shared framework of values. Just as a failure to access the appropriate generalizable scientific evidence can mean that flawed clinical decisions result, so (and perhaps more commonly) a failure to ascertain and work with the values affecting the individual consultation can also result in disaster.

Altamirano-Bustamante and colleagues talk of the axiological complexity of clinical practice which extends beyond the EBM domain of epistemological values related to 'describing, explaining or predicting what takes place within the human body' to the VBM domain of social, political and ethical values acting on 'the bio-psycho-social spheres of a person and relating to his/her dignity' [4]. The pathfinders for EBM were quick to recognize the importance of values in this respect. 'By patient values we mean the unique preferences, concerns and expectations each patient brings to the clinical encounter and which must be integrated (with best research evidence and clinical experience) into clinical decisions if they are to serve the patient' [5].

There is a universal truism in this research group's acknowledgement that 'The healthcare sector is currently facing a crisis of knowledge, compassion, care, cost and values in general; however, few programs have addressed values among healthcare personnel, and little data exist concerning the effectiveness of such programs'. Values are action guiding [6,7]. This is the rationale for exploring how a continuing medical education (CME) program impacts on the way in which healthcare workers operate in respect of EBM and VBM. Can we facilitate clinicians to work better with values in conjunction with evidence?

The stakes are high, because I expect that there is another universal truth in Altamirano-Bustamante *et al*.'s contention that 'patients complain more about the lack of courtesy, warmth, understanding, care, and communication than about the lack of updated attention protocols' [4]. If patients sense that a clinician has worked hard to understand their values and to take account of them, the likelihood of partnership working is greatly increased, and antagonism is less likely. By the same token, the clinician who has understood the values at play is more likely to access the relevant_evidence (for example, what is the point in exploring different psychological treatments for depression if the patient has no belief in any of them and only gives credence to pharmaceutical treatments, or vice versa).

## EBM and VBM: complementary and necessary components of medical decision making

In this impressive study, the authors seized the opportunity to research impact on 973 participants (roughly one-third of those who enrolled for an online course in clinical ethics, accredited for 60 hours CME). The diversity of participants strengthens the findings. With a majority of females (62%), participants' ages ranged widely, and their backgrounds are mixed in terms of profession (57% physicians; 20% nurses) and discipline with 41% working in preventive medicine and primary care, while 54% are in specialist and hospital based services.

The educational intervention used in this study is carefully conceived. For me, the incorporation of real-time decision making is highly important. In the research evaluation, mixed methods are used appropriately to both detect change across the intervention and to go some way towards explaining it. One of the key findings takes a little unpicking. The claim for 'work values' is that CME can 'engineer' strong connections and networks between EBM and VBM. This claim rests on the finding of highly significant increases in mean scores for openness to change (OC) and self-transcendence (ST) after CME intervention. In Schwartz's original value inventory, ten value types are described. Now increased to 19 values, all can be amalgamated into 4 'super groups' of higher-order values. OC and ST are both higher order values. The values comprising openness to change are different from those for self-transcendence [8]. Finding evidence of these axiomatically different 'super group' constructs, the authors claim evidence of networking between evidence basing and values basing. From an educational point of view I find it difficult to justify the claim here. Perhaps I might phrase it differently. The study demonstrates that it is possible for well designed CME to stimulate change that is likely to enhance an individual's practice in both EBM and VBM.

The use of the word 'network' throughout this paper is interesting. Most commonly, 'networks' in the evidence-based practice (EBP) and values-based practice (VBP) literature concern collaborative partnership working by the clinician with others. That is not the case here, where individuals were studied in their use of an online education program. The network concept here is of a conceptual network (rather more like a neural network) being that individuals think about the two pillars of clinical decision making simultaneously and iteratively. In a previous work, this was put as 'think values: think evidence' and 'think evidence: think values' [2] making the point that often clinicians are trapped into thinking unidimensionally. Altamirano-Bustamante *et al*.'s network thinking dispels these unilateral thought processes [4].

Other findings of importance include increased knowledge related to integral ethics [9]. For many clinicians, virtue, duty, and consequence are blurred concepts, and I support the inference that improved understanding of these motivators could improve clinical outcomes for patients. Likewise, there is some evidence to support the claim of personal growth among the participants.

## Future directions and conclusions

Few could argue with the conclusions of this study emphasizing the need for a focus on complementary EBM and VBM. The VBM priorities proposed for CME align well with the clinical skills processes put forward by Fulford *et al. *[2], namely awareness of values; reasoning about values; knowledge about values; and communication skills.

It is worth remembering that the study by Altamirano-Bustamante and colleagues was working from predefined categorization of values. A large part of the study concerned participants ranking from a list.

Real-world values-based practice differs in that for much of the time the values at play are messy and ill defined. To illustrate the point I am making, see Table 3 in Altamirano-Bustamante *et al. *titled 'Values and healthcare personnel roles'. Intended to demonstrate shifts in values over the intervention period, the quotations illustrate some of the complexities of defining values: more often than not there are multiple constructs rolled up in a single values statement. Take the statement about compassion: this reveals something about the healthcare worker's views on religion, psychological intervention; pragmatism (for example, need to earn money); and paternalism (for example, I told the woman to get a job).

This study has given us a very solid foundation on which to build. Moving forward, there is a place for more qualitative educational research regarding how to link EBM and VBM in their clinical practice. It is likely that case studies will play a prominent part in this.

## Competing interests

EP is also researching in the field of education for values-based practice.

## Authors' information

EP is Professor Emeritus (Medical Education), University of Warwick; Visiting Research Professor in the School of Medicine, Dentistry & Biomedical Sciences, Queen's University Belfast; Foundation 'Ronald Harden' Visiting Professor of Medical Education, International Medical University, Malaysia; Editor in Chief, 'Education for Primary Care'. Website at http://www2.warwick.ac.uk/fac/med/study/research/vbp/.

## Pre-publication history

The pre-publication history for this paper can be accessed here:

http://www.biomedcentral.com/1741-7015/11/40/prepub
